# Correction to: Myocardial T1 mapping and extracellular volume quantification: an overview of technical and biological confounders

**DOI:** 10.1007/s10554-018-1327-z

**Published:** 2018-03-09

**Authors:** Stefan K. Piechnik, Michael Jerosch-Herold

**Affiliations:** 1 Oxford Centre for Clinical Magnetic Resonance Research, Division of Cardiovascular Medicine, Radcliffe Department of Medicine, University of Oxford, John Radcliffe Hospital, Oxford, OX39DU UK; 2 Brigham and Women’s Hospital, and Harvard Medical School, 15 Francis Street, Boston, MA 02115 USA

## Correction to: The International Journal of Cardiovascular Imaging (2018) 34:3–14 10.1007/s10554-017-1235-7

The article “Myocardial T1 mapping and extracellular volume quantification: an overview of technical and biological confounders”, written by “Stefan K. Piechnik and Michael Jerosch‑Herold”, was originally published Online First without open access. After publication in volume 34, issue 1, page 3–14, the author decided to opt for Open Choice and to make the article an open access publication. Therefore, the copyright of the article has been changed to © The Author(s) 2018 and the article is forthwith distributed under the terms of the Creative Commons Attribution 4.0 International License (http://creativecommons.org/licenses/by/4.0/), which permits use, duplication, adaptation, distribution and reproduction in any medium or format, as long as you give appropriate credit to the original author(s) and the source, provide a link to the Creative Commons license, and indicate if changes were made.

